# Inhibition of Zn(II) Binding Type IA Topoisomerases by Organomercury Compounds and Hg(II)

**DOI:** 10.1371/journal.pone.0120022

**Published:** 2015-03-23

**Authors:** Bokun Cheng, Thirunavukkarasu Annamalai, Shayna Sandhaus, Priyanka Bansod, Yuk-Ching Tse-Dinh

**Affiliations:** 1 Department of Biochemistry and Molecular Biology, New York Medical College, Valhalla, New York, United States of America; 2 Department of Chemistry and Biochemistry, Florida International University, Miami, Florida, United States of America; 3 Biomolecular Sciences Institute, Florida International University, Miami, Florida, United States of America; University of Oklahoma, UNITED STATES

## Abstract

Type IA topoisomerase activities are essential for resolving DNA topological barriers via an enzyme-mediated transient single strand DNA break. Accumulation of topoisomerase DNA cleavage product can lead to cell death or genomic rearrangement. Many antibacterial and anticancer drugs act as topoisomerase poison inhibitors that form stabilized ternary complexes with the topoisomerase covalent intermediate, so it is desirable to identify such inhibitors for type IA topoisomerases. Here we report that organomercury compounds were identified during a fluorescence based screening of the NIH diversity set of small molecules for topoisomerase inhibitors that can increase the DNA cleavage product of *Yersinia pestis* topoisomerase I. Inhibition of relaxation activity and accumulation of DNA cleavage product were confirmed for these organomercury compounds in gel based assays of *Escherichia coli* topoisomerase I. Hg(II), but not As(III), could also target the cysteines that form the multiple Zn(II) binding tetra-cysteine motifs found in the C-terminal domains of these bacterial topoisomerase I for relaxation activity inhibition. *Mycobacterium tuberculosis* topoisomerase I activity is not sensitive to Hg(II) or the organomercury compounds due to the absence of the Zn(II) binding cysteines. It is significant that the type IA topoisomerases with Zn(II) binding domains can still cleave DNA when interfered by Hg(II) or organomercury compounds. The Zn(II) binding domains found in human Top3α and Top3β may be potential targets of toxic metals and organometallic complexes, with potential consequence on genomic stability and development.

## Introduction

Type IA topoisomerases are present in most organisms to provide the capability of resolving topological barriers that require passage of DNA strand(s) through an enzyme bridged single-strand DNA break [1, 2]. Bacterial topoisomerase I responsible for removal of excess negative supercoiling from chromosomal DNA is the major type IA topoisomerase activity in bacteria, with *Escherichia coli* topoisomerase I as the most extensively studied example. Accumulation of bacterial topoisomerase I covalent intermediate formed between the enzyme and cleaved DNA has been shown to be bactericidal through genetic studies of mutants defective in DNA rejoining, thus validating bacterial topoisomerase I as an attractive novel target for discovery of new antibacterial compounds to combat multi-drug resistant bacterial pathogens [3]. Topoisomerase poison inhibitors that inhibit the catalytic step of DNA rejoining by type IB human topoisomerase I or type IIA human and bacterial topoisomerases are clinically important anticancer and antibacterial agents [4]. Such topoisomerase poison inhibitors have the advantage that a small number of topoisomerase cleavage complexes stabilized on the chromosome may be sufficient for initiation of bacterial cell death as shown for quinolones acting as *E*. *coli* DNA gyrase poison inhibitors [5]. A fluorescence based assay adaptable for high throughput screening was developed for identification of similar poison inhibitors for type IA topoisomerases that can increase the oligonucleotide cleavage product accumulation for bacterial topoisomerase I [6]. We report here that during assay development and pilot screening of NIH Diversity Set I compounds against *Yersinia pestis* topoisomerase I, organomercury compounds were identified as assay hits. Results shown here from additional biochemical experiments demonstrated that organomercury compounds and mercury chloride target the cysteines present in the C-terminal domains of *E*. *coli* and *Y*. *pestis* topoisomerase I for inhibition of relaxation activity. These results have significant implications both for the discovery of bacterial topoisomerase I inhibitors, and the potential vulnerability of human type IA topoisomerases to inhibition by toxic metal and organometallic compounds.

## Materials and Methods

### Topoisomerase Enzymes

Recombinant *Y*. *pestis*, *E*. *coli* and *M*. *tuberculosis* DNA topoisomerase I enzymes were expressed in *E*. *coli* and purified with previously described procedures [7–9].

### Compounds

NIH diversity set I (1990 compounds with information on the set available in http://dtp.nci.nih.gov/branches/dscb/diversity_explanation.html) and individual compounds from the set were provided by the NCI/DTP Open Chemical Repository (http://dtp.cancer.gov). Individual compounds were suspended in dimethyl sulfoxide (DMSO) at a concentration of 40 mM. The compound solutions were stored at -30°C as working solutions or at -80°C for long term.

### Fluorescence assay for increase in bacterial topoisomerase I cleavage product

**Fig 1:**
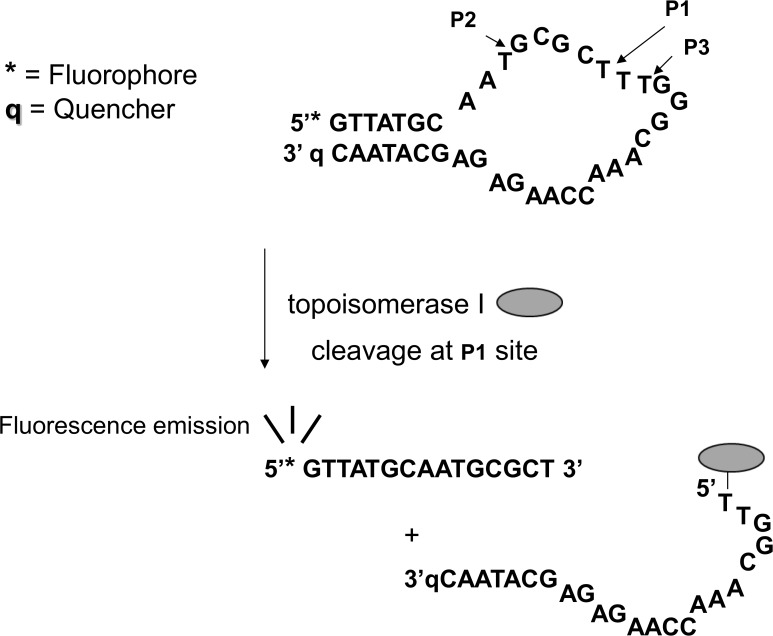
Scheme of fluorescence assay to identify compounds that can increase the level of DNA cleavage product from bacterial topoisomerase I due to increase in fluorescence emission from fluorophore. The structure shown is predicted by mfold [10], with constraints for the first and last bases be paired to quench the emission from the fluorophore, and the cleavage sites P1, P2, P3 be in single-stranded region of the structure.

The oligonucleotide substrate with sequence (5’-GTTATGCAATGCGCTTTGGGCAAACCAAGAGAGCATAAC-3’) was designed to produce an increase in fluorescence signal with a higher level of bacterial topoisomerase I cleavage. Fluorescence emission from a fluorophore placed at the 5’-end is limited by the presence of a quencher at the 3’-end due to a short-stem secondary structure (Fig. 1). Melting of the secondary structure at high temperatures corresponds to an increase of fluorescence by 16-fold. Cleavage by topoisomerase I in the loop region at three potential sites (P1, P2, P3) would destabilize the stem structure and increase the fluorescence signal as well [6]. P1 was found to be the most preferred cleavage site for *E*. *coli* topoisomerase I. For initial assay development, the FAM fluorophore was placed at the 5’-end and BHQ-1 quencher was placed at the 3’-end by custom synthesis (Sigma Genosys). Screening of the NIH diversity set I compounds was carried out in 96-well black plate. The reaction mixture of 100 nM oligo substrate, 100 nM *Y*. *pestis* topoisomerase I in 10 mM Tris-HCl (pH 8) and 0.5 mM MgCl_2_ were dispensed in 100 μl aliquots, followed by addition of 1 μl of 10 mM compounds or 1 μl DMSO as negative control. Following 30 min at 37°C, fluorescence (Ex/Em wavelengths of 485/528 nm) was recorded with the BioTek Synergy HT plate reader. The assay on the positive compounds was later repeated using an oligonucleotide substrate with CAL Fluor Red 610 fluorophore at the 5’-end and BHQ-2 quencher at the 3’-end supplied by Biosearch Technologies (Ex/Em wavelengths of 590/610 nm) to eliminate false positive signals from compounds with autofluorescence in the same wavelength range as FAM. For follow up dose-response experiments, compound concentrations varied between 50–800 μM with DMSO concentration kept at 1%.

### Gel based assay of oligonucleotide DNA cleavage product accumulation

The oligonucleotide substrate without the fluorophore or quencher was labeled at the 5’-end with γ-^32^P-ATP and T4 polynucleotide Kinase. The labeled oligonucleotide (0.5 pmole) was incubated with 100 ng of *Y*. *pestis* or *E*. *coli* topoisomerase I in 5 μL of 10 mM Tris-HCl, pH 8.0, in the presence or absence of 2 mM MgCl_2_. Compound solutions or control DMSO was added in 0.25 μl volumes. After incubation at 37°C for 30 min the reaction was terminated by the addition of an equal volume of sequencing gel loading buffer. The reaction substrate and products were separated by electrophoresis in a 15% sequencing gel followed by Phosphor-Imager analysis of the gel.

### Relaxation activity assay

Supercoiled pBAD/Thio plasmid DNA substrate was purified by CsCl gradient centrifugation. The relaxation reaction was carried out in 10 mM Tris-HCl, pH 8.0, 50 mM NaCl, 0.1 mg/mL gelatin and 0.5 mM MgCl_2._ Compounds or DMSO (0.5 μl volume) were added to 10 μl of the reaction mixture containing 250 ng of supercoiled DNA before combining with 10 μl of the reaction mixture containing either 20 ng of *E*. *coli* topoisomerase I or 50 ng of *Y*. *pestis* topoisomerase I enzyme to initiate the relaxation reaction. Following incubation at 37°C for 30 min, the reactions were terminated and analyzed by agarose gel electrophoresis as previously described [11].

### Non-covalent DNA binding assay

Non-covalent DNA binding by *E*. *coli* topoisomerase I was measured by a fluorescence anisotropy assay as described previously [12] in binding buffer of 50 mM Tris–HCl, pH 7.5, 100 mM NaCl, 0.1 mM EDTA, 0.5 mM MgCl_2_, with a 59-base oligonucleotide 5’-GCCCTGAAAGATTATGGAATGGGATTAGGGTAAAGGAAGAGAGCATAATCTTTCAGGGC-3’ that also forms a stem-loop structure and has a 6-carboxyfluorescein modification at the 3’-end (supplied by Biosearch Technologies). This substrate was shown in a previous report to bind with high affinity to *E*. *coli* topoisomerase I, but cannot be cleaved by the enzyme due to the absence of a cytosine in the single-stranded DNA region [12].

## Results

### Increase in fluorescence signal from organomercury compounds identified in pilot screening of NIH Diversity Set I

**Fig 2:**
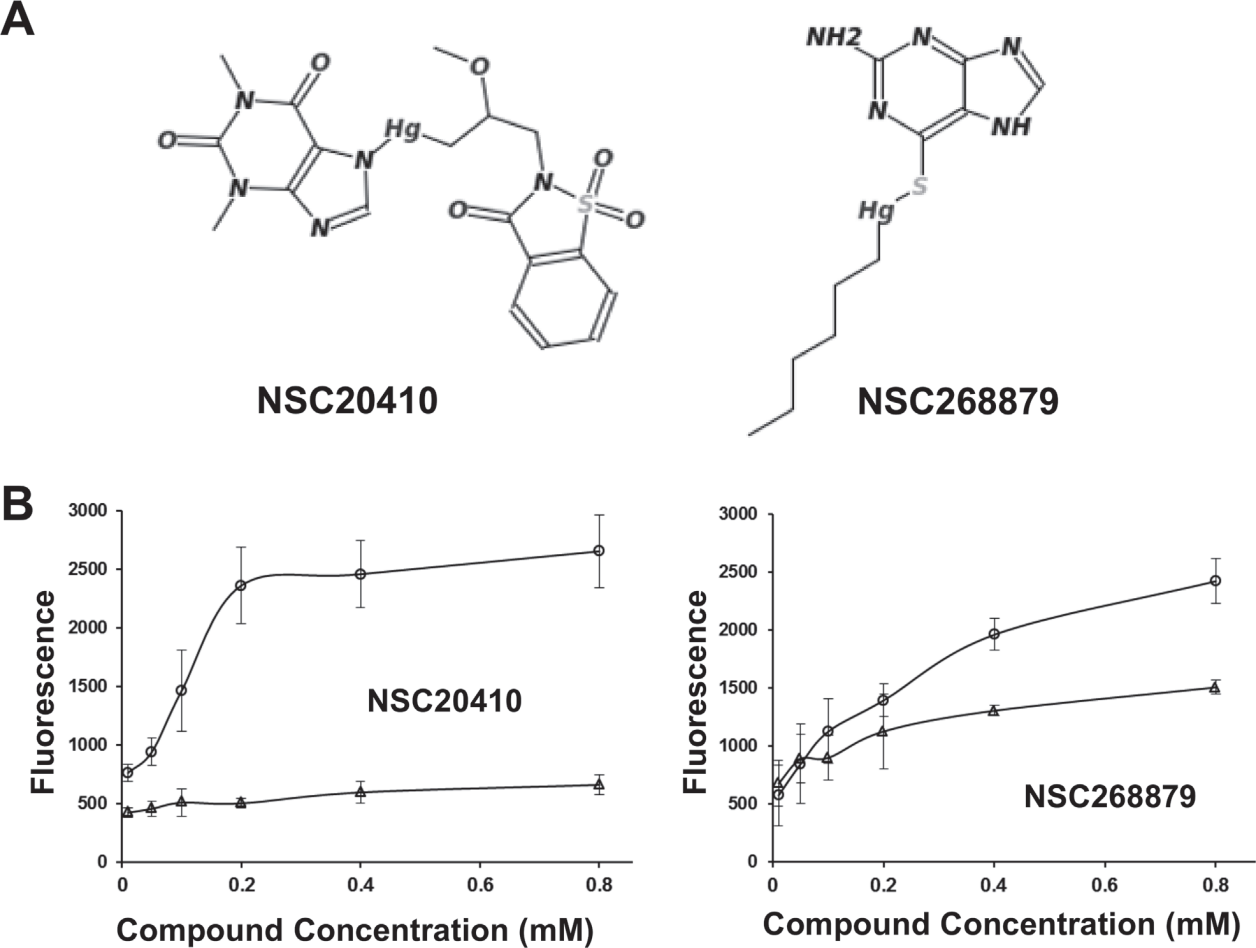
Organomercury hits from fluorescence based assay. A. Structures of organomercury hits from fluorescence based assay for increase in bacterial topoisomerase I mediated oligonucleotide cleavage. B. Dose response of increase fluorescence reading (in arbitrary units) upon addition of the two hit compounds (0.05–0.8 mM) in the absence (Δ) and presence (O) of *Y*. *pestis* topoisomerase I. Error bars correspond to standard deviations from at least three sets of data.

A high-throughput screening assay was designed to identify inhibitors that can act as topoisomerase poisons for type IA topoisomerases. This assay utilizes an oligonucleotide substrate with a 5’-fluorophore quenched by a 3’-quencher to produce an increase in fluorescence signal from a higher level of oligonucleotide cleavage product produced by bacterial topoisomerase I from the action of topoisomerase poison inhibitors. *Y*. *pestis* topoisomerase I was used in the assay development due to its potential significance for bioterror concerns. The NIH Diversity Set I of 1990 compounds was tested to identify potential positive control compounds during assay development. Among the positive hits (compounds that increase the fluorescence signal by 2 fold over DMSO negative control), there were two organomercury compounds, NSC20410 and NSC268879 (Fig. 2A). Dose response of the effect of these compounds on the increase in fluorescence signal were evaluated both in the presence of enzyme or absence of enzyme as a counter assay in order to eliminate compounds that did not produce enzyme-dependent increase in fluorescence. The results (Fig. 2B) showed that the dose-dependent increase in fluorescence signal from the addition of NSC20410 is entirely dependent on the presence of the topoisomerase I enzyme. Compound NSC268879 could cause an increase of fluorescence in the absence of enzyme, but the level of fluorescence signal was further increased in the presence of enzyme.

### Inhibition of type IA topoisomerase relaxation activity by organomercury compounds is dependent on presence of Zn(II)-binding cysteines

**Fig 3:**
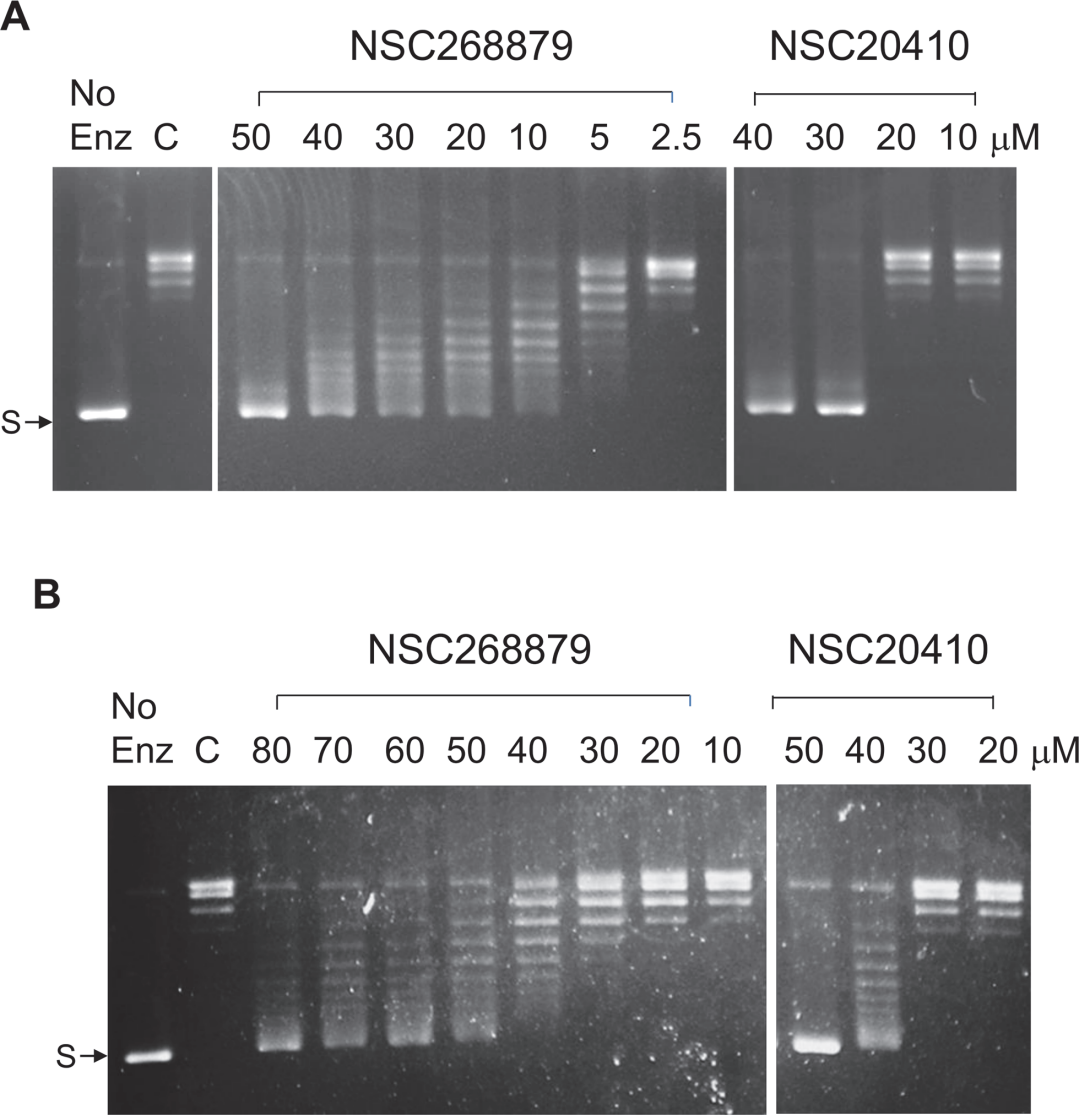
Inhibition of topoisomerase I relaxation activity by the organomercury compounds. A. *Y*. *pestis* topoisomerase I. B. *E*. *coli* topoisomerase I. Assay was carried out in the presence of DMSO (C) or the indicated concentrations of compound added to the reaction mixture before the addition of enzyme. No enz: no enzyme added. S: supercoiled pBAD/Thio DNA.

The relaxation activity of *Y*. *pestis* topoisomerase I was assayed using supercoiled pBAD/Thio plasmid DNA as the substrate. Inhibition of the relaxation activity was observed as expected (Fig. 3A). The IC50 for inhibition is between 20–30 μM for NSC20410 and is approximately 20 μM for NSC286679. Similar results (IC50 between 40–50 μM) were obtained for *E*. *coli* topoisomerase I (Fig. 3B).

**Fig 4:**
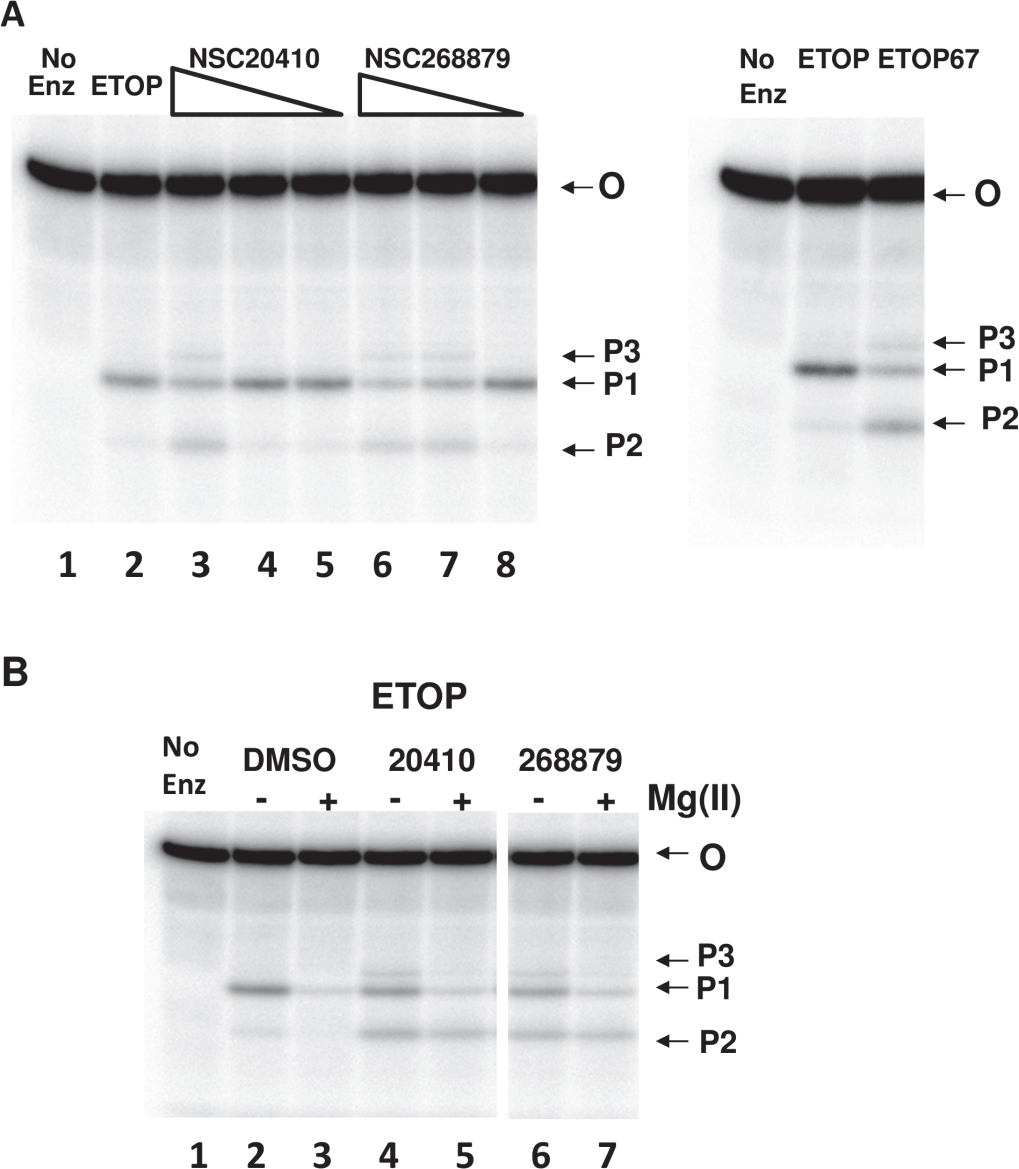
Effect of NSC20410 and NSC268879 on oligonucleotide cleavage product accumulation by *E*. *coli* topoisomerase I. A. Cleavage products (P1, P2, P3) formed in the absence of Mg(II). *E*. *coli* topoisomerase I (ETOP) or its 67 KD N-terminal domain (ETOP67) was incubated with 5’-^32^P labeled oligonucleotide substrate (O). Left panel: lane 1: substrate only control with no enzyme, lane 2: substrate incubated with ETOP in the presence of DMSO. NSC20410 (lanes 3–5) and NSC268879 (lanes 6–8) were included in ETOP reactions at concentrations of 100, 10, 2 μM. Right panel: Comparison of cleavage products formed by ETOP and ETOP67 with no inhibitors present. B. Inhibition of Mg(II)-mediated religation of DNA cleavage products NSC20410 and NSC268879. Products accumulated in the absence (-) or presence (+) of 2 mM MgCl_2_ were compared with control DMSO (lanes 2,3), 100 uM NSC20410 (lanes 4,5) or 10 uM NSC268879 (lanes 6,7) added.

The increased accumulation of DNA cleavage product, as indicated by the fluorescence assay, was confirmed for *E*. *coli* topoisomerase I with a gel-based assay using the same oligonucleotide sequence from the fluorescence assay with the ^32^P-label introduced at the 5’-end of the oligonucleotide substrate (Fig. 4). Analysis of the formation of the 5’-end labeled DNA cleavage reaction products showed that the presence of the organomercury compounds did not inhibit the DNA cleavage step, but rather resulted in an overall increase in DNA cleavage (Fig. 4A). In the absence of Mg(II), product P1 (5’-GTTATGCAATGCGCT) is the only major product observed from the cleavage of the substrate by *E*. *coli* topoisomerase I, while P2 is a very minor product (5’-GTTATGCAAT). The addition of the organomercury compounds resulted in the significant increase of the cleavage product P2 (up to 8-fold increase from NSC20410 and 6-fold increase from NSC268879 when quantitated by densitometry analysis) and appearance of another minor cleavage product P3 (5’ GTTATGCAATGCGCTTT), slightly longer in length than product P1. This is consistent with the overall increase in DNA cleavage product formation, with the shortest cleavage product, P2, being observed most readily. If cleavage takes place at more than one site on a 5’-end labeled DNA substrate, then only the labeled product corresponding to the cleavage site closest to the 5’-end will be observed [13, 14]. Interestingly, the effect of these two organomercury compounds is similar to the effect of removal of the 30 kDa C-terminal domain from the protein sequence as shown by the cleavage products formed by ETOP67 (right hand panel of Fig. 4A), the 67 kDa N-terminal fragment of *E*. *coli* topoisomerase I [15]. The presence of Mg(II) shifts the DNA cleavage-religation equilibrium of *E*. *coli* topoisomerase I towards DNA religation, decreasing the amount of cleavage products. This is evident from the densitometry analysis of the results shown in Fig. 4B, where in the presence of Mg(II) (lane 3), cleavage products (P1, P2) are at 4% of the total oligonucleotide substrate in comparison to 19% in the absence of Mg(II) (lane 4). However, densitometry analysis showed that the combined levels of the three cleavage products (P1, P2, P3) remaining following incubation with Mg(II) were 3-fold higher in the presence of the inhibitors NSC20410 (lane 5) or NSC268879 (lane 7) versus the DMSO control (lane 3). This indicates that the organomercury compounds inhibited DNA religation by *E*. *coli* topoisomerase I when Mg(II) was present. The inhibition of religation of P2 cleavage product was observed most readily since it corresponds to the cleavage site closest to the 5’-end label site.

**Fig 5:**
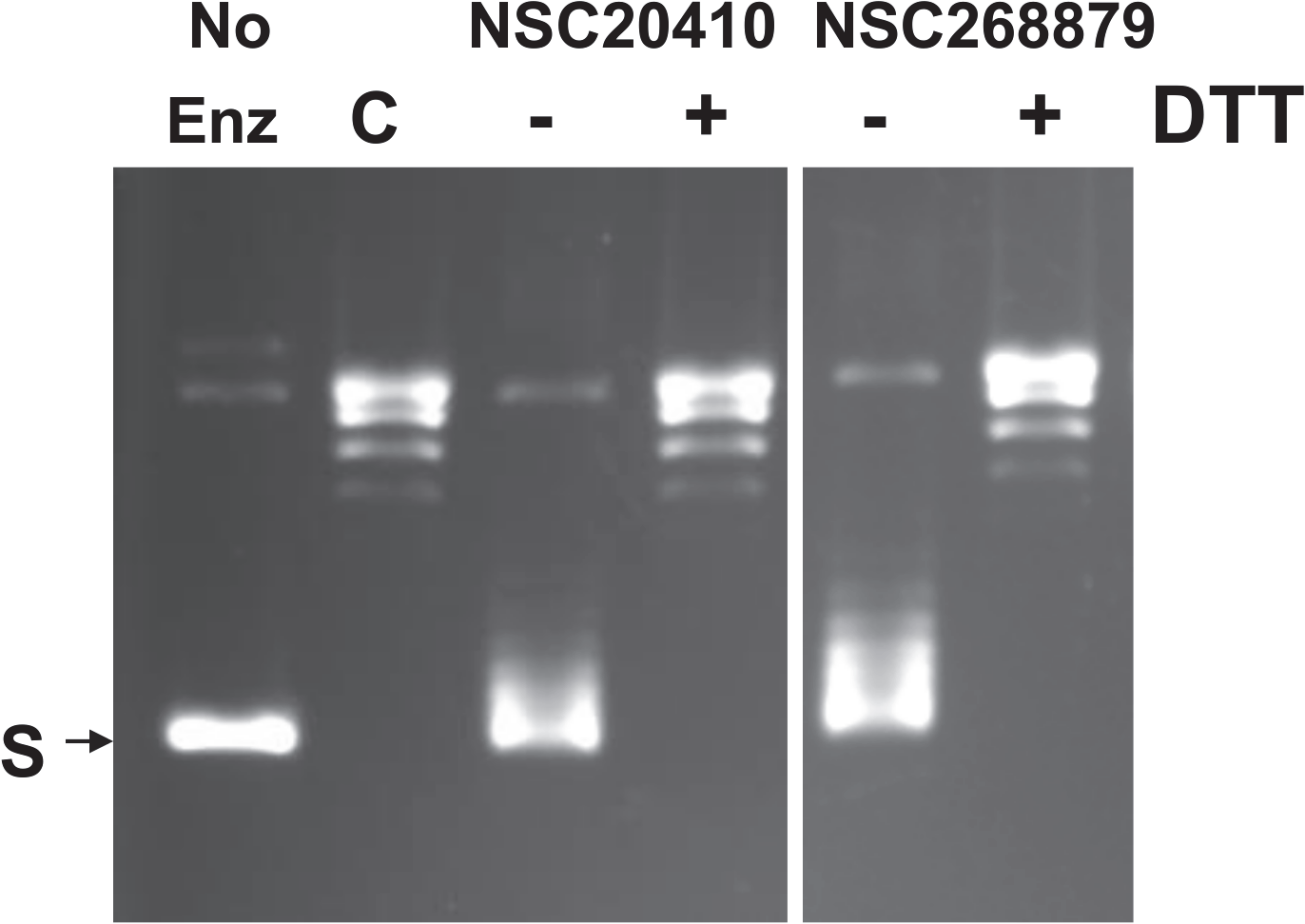
Protection of topoisomerase relaxation activity inhibition by DTT. No Enz: no enzyme. *E*. *coli* topoisomerase I (50 ng) were present in all the other lanes. C: DMSO control. Compounds and DTT were present at 50 μM. S: supercoiled pBAD/Thio plasmid DNA.

The 30 kDa C-terminal domain of *E*. *coli* and *Y*. *pestis* topoisomerase I has three tetra-cysteine zinc ribbon motifs that each bind a Zn(II) [16]. The Zn(II) ions can be replaced by Cd(II) but not with other metal ions without loss of overall relaxation activity [17, 18]. Recombinant *E*. *coli* topoisomerase with the zinc replaced by iron has been characterized and found to have no activity on a DNA substrate [19]. The bacterial topoisomerase I enzymes share a highly homologous 67 kDa N-terminal transesterification domain [20]. However, the C-terminal domain can diverge significantly among the topoisomerase I enzymes from the different bacterial species [20]. The topoisomerase I enzymes from Mycobacteria species have evolved to have a C-terminal domain that lacks the zinc-binding tetra-cysteine motifs in *E*. *coli* and *Y*. *pestis* topoisomerase I [21, 22]. The two organomercury compounds were found to have no effect on the relaxation activity of *M*. *tuberculosis* topoisomerase I (S1 Fig.). Increase in fluorescence was also not observed in the fluorescence-based oligonucleotide cleavage assay using *M*. *tuberculosis* topoisomerase I. Dithiothreitol (DTT) was found to protect the relaxation activity of *E*. *coli* topoisomerase I from inhibition by these organomercury compounds (Fig. 5). Therefore, the cysteine residues that form the Zn(II)-binding motifs in the *E*. *coli* and *Y*. *pestis* topoisomerase I are the likely target for the organomercury compounds identified in the screening assay.

### Inhibition of relaxation activity by Hg(II) but not As(III)

**Fig 6:**
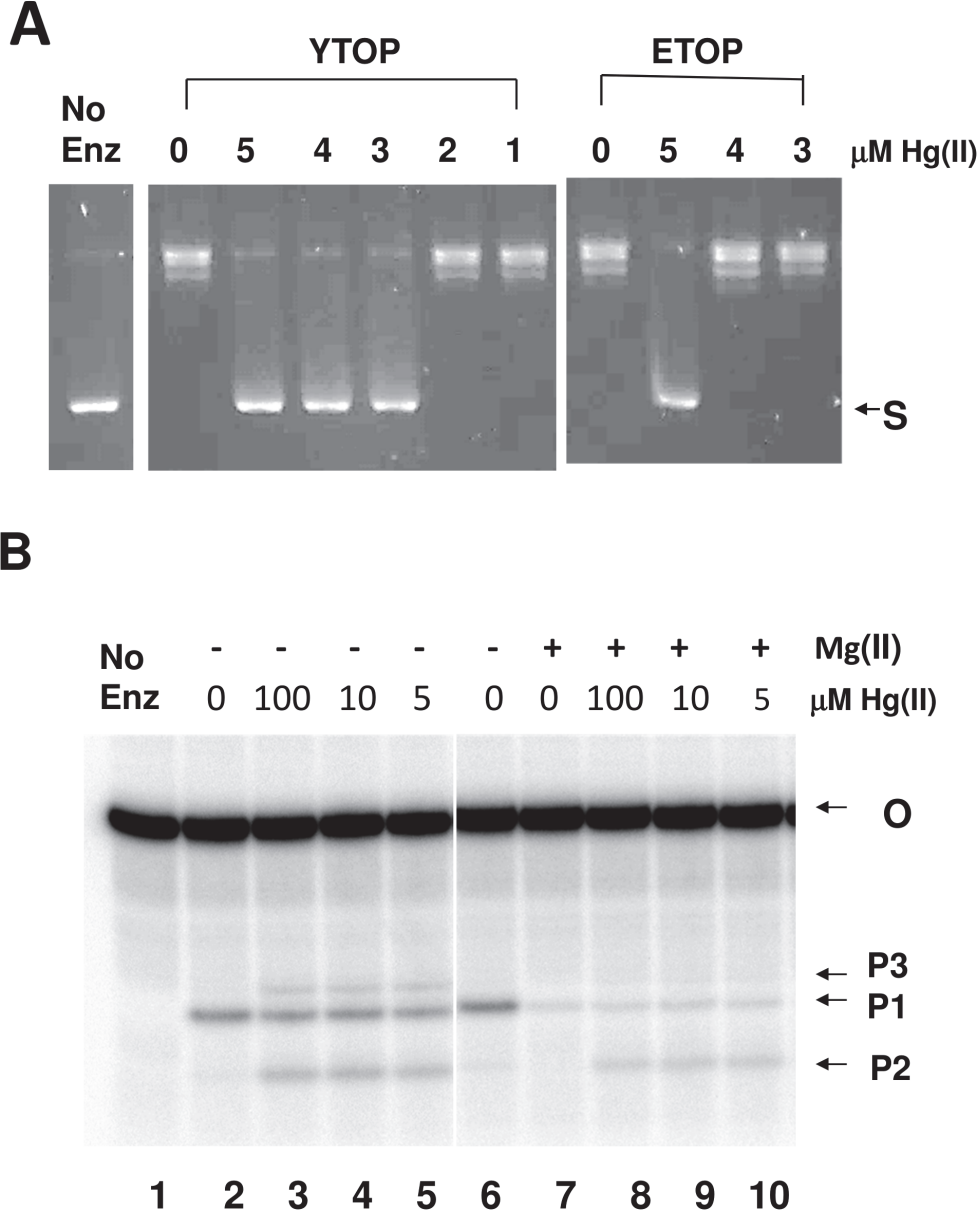
Effect of mercury chloride on *Y*. *pestis* and *E*. *coli* topoisomerase I activity. A. Assay of inhibition of relaxation activity of *Y*. *pestis* (YTOP) and *E*. *coli* topoisomerase I (ETOP). S: Supercoiled pBAD/Thio plasmid DNA. B. Mercury chloride does not abolish DNA cleavage by ETOP and increases the yield of the shortest ETOP cleavage product both in the absence and presence of Mg(II). Oligonucleotide substrate (O) and cleavage products (P1, P2, P3) from *E*. *coli* topoisomerase I with no MgCl_2_ (lanes 2–6), or 2 mM MgCl_2_ (lanes 7–10) were analyzed. Lane 1: no enzyme control. HgCl_2_ is present at 0 μM (lanes 2,6,7), 5 μM (lanes 5, 10), 10 μM (lanes 4, 9) and 100 μM (lanes 3, 8). The lanes shown are from the same PhosphorImager gel record.

**Fig 7:**
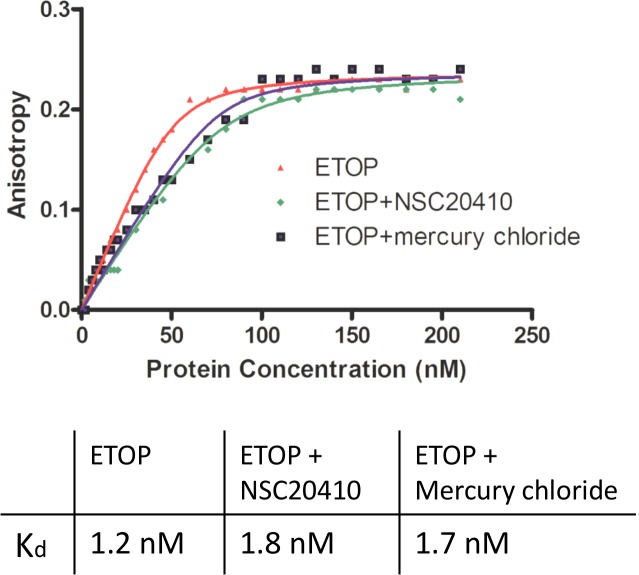
Noncovalent DNA binding by *E*. *coli* topoisomerase I in the absence and presence of NSC20410 and mercury chloride as determined by DNA fluorescence anisotropy. Graphs showing anisotropy change observed when DNA substrate with 6-carboxyfluorescein modification at 3’-end (30 nM) was titrated with increasing concentrations of *E*. *coli* topoisomerase I in the absence of inhibitor (red triangles), or in the presence of 45 μM NSC20410 (green circles), or 5 μM mercury chloride (blue squares). The K_d_ (dissociation constant) values were calculated from fitting of the anisotropy data from 10, 20 and 30 nM oligonucleotide concentrations as described previously [12].

Inorganic Hg(II) was also tested to see if it has a similar effect on these type IA topoisomerases on its own without the bound organic ligand. Mercury chloride was found to inhibit the relaxation activity of both *Y*. *pestis* and *E*. *coli* topoisomerase I (Fig. 6A). In contrast, another known zinc finger protein interacting metal such as Arsenic (III) [23] in the form of sodium meta arsenite (AsNaO_2_) at concentrations up to 10 mM did not have any effect on the relaxation activity of *E*. *coli* topoisomerase I (S2 Fig.). Mercury chloride inhibited *Y*. *pestis* topoisomerase I slightly more than *E*. *coli* topoisomerase I, similar to the results observed for the organomercury compounds. Hg(II) also affected oligonucleotide cleavage by *E*. *coli* topoisomerase I in similar manners as the organomercury compounds. Hg(II) increased the relative level of the shortest 5’-end labeled cleavage product P2 over the longer cleavage product P1 (Fig. 6B). Similar to the organomercury compounds, densitometry analysis showed that in the absence of Mg(II) there is a significant increase in the shortest cleavage product P2 in lanes 3–5 (up to 10-fold when compared with the lane 2 containing no compound). Also, similar to results shown in Fig. 4B there is a shift towards DNA religation in the presence of 2 mM Mg (II), resulting in lesser combined cleavage products in lane 7 compared to lane 6 without Mg (II). HgCl_2_ concentrations between 5–100 μM, when incubated with Mg (II), resulted in up to 2-fold increase in combined cleavage products (lanes 8–10) when compared to the no HgCl_2_ reaction (lane 7). This result indicates that Hg(II) preferentially inhibits DNA religation by these type IA topoisomerases and increases the level of DNA cleavage. The increase in DNA cleavage caused by NSC20410 and Hg(II) is not due to increase in DNA binding affinity. The measurement of non-covalent binding by an anisotropy assay (Fig. 7) showed that the presence of NSC20410 or HgCl_2_ had only a minor effect on the non-covalent binding to DNA, with nearly identical maximal anisotropy and a less than 1-fold effect on the dissociation constant Kd.

## Discussion

Bacterial topoisomerase I is a potentially useful target for the discovery of much needed new leads for antibacterial therapeutics that can be used to treat drug resistant pathogens [3]. Topoisomerase poison inhibitors that can increase the DNA cleavage intermediates formed by type IA topoisomerases with the required selectivity still remain to be identified [4]. Such type IA topoisomerase poison inhibitors are expected to be bactericidal based on the study of bacterial topoisomerase I mutants deficient in DNA religation following DNA cleavage [7, 24]. During assay development of a HTS assay for identifying potential poison inhibitors against *Y*. *pestis* topoisomerase I, two organomercury compounds in the NIH Diversity Set I of compounds were found in the pilot screening to increase the level of DNA cleavage products formed by *Y*. *pestis* topoisomerase I. Gel-based assays confirmed that these compounds did not prevent oligonucleotide DNA cleavage, but rather increased the level of the accumulation of the DNA cleavage products. These two compounds inhibited the relaxation activity of *E*. *coli* and *Y*. *pestis* topoisomerase I, but had no effect on the activity of *M*. *tuberculosis* topoisomerase I, which does not have Zn(II)-binding tetra-cysteine motifs for the C-terminal domain function [22]. The protective effect of DTT, and similar inhibitory actions of inorganic mercury chloride indicated that the zinc-binding cysteines in *E*. *coli* and *Y*. *pestis* topoisomerase I are vulnerable to interference by organomercury and inorganic mercury compounds. The IC50s for inhibition by Hg(II) are about ten-fold lower than the IC50s for the organomercury compounds. The topoisomerase I cysteines in the C-terminal may be more accessible to Hg(II) than the organomercury compounds. Furthermore, NSC20410 had little effect on the DNA substrate fluorescence in the absence of enzyme in contrast to NSC268879 which can result in an increase of fluorescence in the absence of enzyme (Fig. 2B). Hg(II) and NSC20410 are likely to target with binary selectivity the Zn(II)-binding cysteines in the C-terminal domain of the enzyme for coordination by Hg(II), while NSC268879 might interact with both the enzyme and DNA in its mechanism of action. This difference in mechanism of inhibition may account for the more gradual relaxation inhibition observed as NSC268879 concentration increases (Fig. 3).

Mutations of bacterial topoisomerase I shown previously to inhibit DNA religation were all found in the N-terminal domain at the Mg(II) binding TOPRIM residues [7, 24] or surrounding the active site tyrosine [8, 25]. The C-terminal domains of type IA topoisomerases are known to play critical roles in interactions with DNA and other cellular proteins relevant to their catalytic activities and physiological functions. The Zn(II)-binding domain of *E*. *coli* topoisomerase I [15] has been postulated to interact with DNA to promote strand passage for the relaxation of negative supercoils. Site-directed mutations in the Zn(II) coordinating cysteines of *E*. *coli* topoisomerase I have been shown to affect DNA cleavage sequence selectivity and can lead to a significant loss of relaxation activity [26]. The Zn(II)-binding domain of *E*. *coli* topoisomerase I has also been shown to be important for direct protein-protein interaction with the RNA polymerase β’ subunit [27] for relief of transcription-driven supercoiling. This protein-protein interaction helps to prevent inhibition of transcription elongation due to hypernegative supercoiling and resulting R-loops of RNA-DNA hybrids [28]. The C-terminal domain of Drosophila topoisomerase IIIα, a type IA topoisomerase with multiple Zn(II)-binding CCCC motifs, is required for the function of the enzyme in double Holliday junction resolution [29].

It should be noted that the organomercury compounds and Hg(II) did not inhibit the DNA cleavage step of the Zn(II)-binding type IA topoisomerases tested, but rather affected the subsequent step of DNA rejoining. Accumulation of topoisomerase cleavage complexes from the action of topoisomerase poison inhibitors has been linked to genomic translocations and carcinogenesis [30]. Human type IA topoisomerases, including human Top3α and Top3β, play important roles in genomic stability [31] and neuronal development respectively [32, 33] as part of multi-protein complexes that interact with DNA as well as RNA. Human Top3α and Top3β each have multiple units of CCCC zinc ribbon motifs in their C-terminal domains. Trivalent As(III) has been shown to interact selectively with zinc finger proteins containing CCCH or CCCC motifs [23] that accounts, at least in part, for the carcinogenic and toxic effect of arsenite [34]. As(III) however had no effect on the activity of the CCCC zinc ribbon motifs of the bacterial type IA topoisomerases tested here, probably due to the difference in coordination chemistry and geometry. Organomercury or Hg(II) may have adverse effects on protein-protein and protein-DNA interactions of involving similar CCCC zinc ribbon motifs in the C-terminal domains of human type IA topoisomerases. There might be harmful consequence on human health if covalent complexes between topoisomerase and cleaved DNA become stabilized during chromosomal DNA cleavage-rejoining of the topoisomerase catalytic cycle.

## Conclusions

Organomercury compounds and inorganic mercury ions can inhibit the relaxation activity of type IA bacterial topoisomerases with Zn(II)-binding cysteines in their C-terminal domains and lead to the accumulation of DNA cleavage products.

## Supporting Information

S1 Fig Organomercury compounds had no significant effect on the relaxation activity of *M*. *tuberculosis* topoisomerase I. (PDF)

S2 Fig Testing of As(III) for inhibition of *E*. *coli* topoisomerase I relaxation activity. (PDF)
