# Supplementary figures and images for: Inhibition of Zn(II) Binding Type IA Topoisomerases by Organomercury Compounds and Hg(II)
Source: PLoS One. 2015 Mar 23;10(3):e0120022. doi: 10.1371/journal.pone.0120022 (PMC4370478; doi:10.1371/journal.pone.0120022)

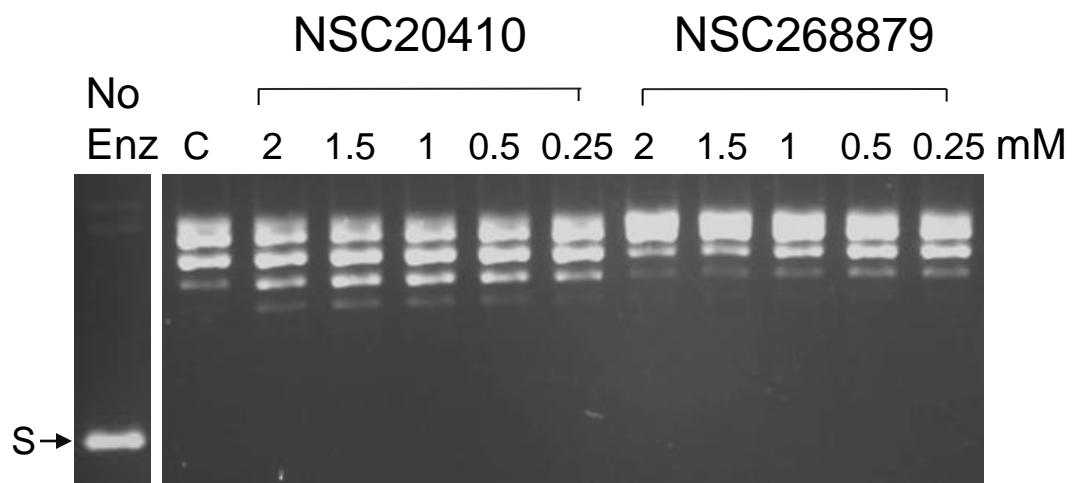

Supplement: S1 Fig — (PDF) [file pone.0120022.s001.pdf]

No

Enz 0 0 1 2 3 4 5 6 7 8 9 10 mM

S →

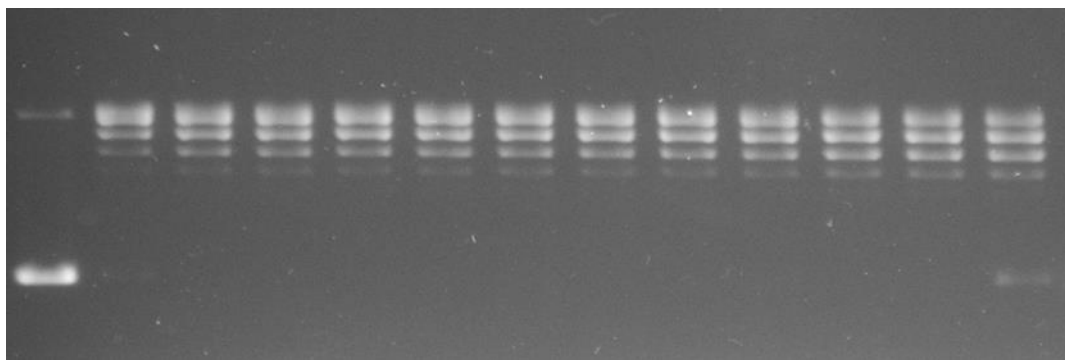

Supplement: S2 Fig — (PDF) [file pone.0120022.s002.pdf]
